# Harmonization of diffusion MRI on healthy subjects using NeuroCombat and LongCombat: a B-Q MINDED brain intra- and inter-scanner study

**DOI:** 10.3389/fnins.2025.1591169

**Published:** 2025-06-03

**Authors:** Maíra Siqueira Pinto, Vincenzo Anania, Roberto Paolella, Céline Smekens, Thibo Billiet, Thomas Janssens, Arnold J. den Dekker, Jan Sijbers, Pieter-Jan Guns, Pieter Van Dyck

**Affiliations:** ^1^Department of Radiology, University Hospital Antwerp (UZA), Antwerp, Belgium; ^2^imec-Vision Lab, University of Antwerp, Antwerp, Belgium; ^3^icometrix, Leuven, Belgium; ^4^Siemens Healthcare NV/SA, Groot-Bijgaarden, Belgium; ^5^μNEURO Research Center of Excellence, University of Antwerp, Antwerp, Belgium; ^6^Department of Physiopharmacology, University of Antwerp, Antwerp, Belgium; ^7^MIRA, Faculty of Medicine and Health Sciences, University of Antwerp, Antwerp, Belgium

**Keywords:** harmonization, diffusion MRI, brain MRI, B-Q MINDED, intra-scanner, inter-scanner, normalization, ComBat

## Abstract

The structural integrity of brain white matter is commonly assessed using quantitative diffusion metric maps derived from diffusion MRI (dMRI) data. However, in multi-site, multi-scanner studies, variability across and within scanners presents challenges in ensuring consistent and comparable diffusion evaluations. This study assesses the effectiveness of ComBat-based harmonization algorithms in reducing intra- and inter-scanner variability in diffusion metrics such as FA, MD, AD, RD, MK, AK, and RK. Utilizing the B-Q MINDED dataset, which includes anatomical and dMRI data from 38 healthy adults scanned twice on two 3T MRI scanners (Siemens Healthineers PrismaFit and Siemens Healthineers Skyra) on the same day, we evaluated the NeuroCombat and LongCombat algorithms for harmonizing diffusion metrics. These harmonization methods effectively minimized both intra- and inter-scanner variability, highlighting their potential to improve consistency in multi-scanner diffusion analysis. Our findings suggest that NeuroCombat and LongCombat are recommended for harmonizing dMRI metric maps in clinical studies. Additionally, both algorithms applied in either ROI-based or voxel-wise configurations, significantly reduced variability, achieving levels comparable to scan-rescan variability intra-scanner. Nonetheless, the choice of harmonization algorithm and implementation should be tailored to the research question at hand. Moreover, the significant intra- and inter-subject variability on non-harmonized diffusion data demonstrated in this study reinforces the importance of harmonization strategies that address any sources of variability. By minimizing scanner-specific biases, the NeuroCombat and LongCombat harmonization algorithms enhance the reliability of diffusion biomarkers, enabling large-scale studies and more informed clinical decision-making in brain-related conditions.

## 1 Introduction

Magnetic resonance imaging (MRI) is widely used in clinical studies thanks to its excellent soft tissue contrast, imaging flexibility, and non-ionizing acquisition. Diffusion MRI (dMRI) is an MRI technique whose contrast is related to the diffusion of water molecules inside tissues, being a powerful technique to probe the human brain microstructure. Nonetheless, dMRI is known to be majorly affected by differences in equipment hardware, software and acquisition parameters, such as differences in pulse sequences, signal-to-noise ratio, image intensity non-uniformity, ghosting artifacts, geometrical distortions, all which cause undesirable variability between dMRI metrics across scanners (Pinto et al., 2020; Jones, 2010; Peltonen et al., 2020; Vollmar et al., 2010). Notably, these variabilities can be in the same order of magnitude as biological variabilities, which severely hinders the interpretation of clinical studies, especially when evaluating quantitative diffusion metrics (Grech-Sollars et al., 2015; Nencka et al., 2018; Pinto et al., 2020; Vollmar et al., 2010).

Standard pre-processing steps in diffusion MRI—such as motion correction, eddy-current correction, distortion correction, and denoising—are essential for addressing within-scan artifacts, but they do not resolve the substantial variability introduced by differences across MRI scanners. This inter-scanner variability stems from hardware-dependent factors like gradient nonlinearities, RF coil sensitivity, and noise profiles; vendor-specific reconstruction algorithms; inconsistencies in b-value implementation and diffusion direction encoding; and temporal or thermal instability affecting scanner performance. Additionally, assumptions embedded in pre-processing tools may not generalize well across different systems, leading to residual bias in derived diffusion metrics such as fractional anisotropy or mean diffusivity. As a result, even with identical acquisition protocols, data acquired across scanners may not be directly comparable, highlighting the need for additional methods beyond pre-processing to ensure consistency in multi-site diffusion MRI studies.

Since the success of a joint analysis of multi-site dMRI data requires data comparability, harmonization of such data is highly recommended (Richter et al., 2022). Computational methods to harmonize the dMRI data and minimize the imaging variability are essential/critical to reliably combine datasets acquired from different scanners and/or protocols, thus improving the statistical power and sensitivity of multi-scanner studies. A variety of computational approaches have been proposed to harmonize dMRI data and remove scanner-specific effects that can affect the data interpretation. However, harmonizing dMRI images comes with a few drawbacks, including the need to share images across sites (data sharing, anonymization, and data volume challenges), and a priori requirements for study design (e.g., participants to be matched in age and gender across sites, or protocols to be consistent across sites) (Richter et al., 2022). While performing dMRI harmonization, one needs to consider the harmonization factors at the acquisition level (hardware and software settings) and the harmonization factors at the image level (raw dMRI data or diffusion metric maps). To reduce complexity, harmonizing 3D diffusion metric maps (e.g., FA, MD, and MK) compared to 4D raw dMRI data, is often more feasible.

In recent years, a variety of statistical and machine learning methods have been proposed to address the challenge of multi-site and multi-scanner variability in neuroimaging data. Traditional approaches, such as linear mixed-effects (LME) models, enable the modeling of both fixed and random effects and have been applied to account for site and scanner differences while preserving biologically relevant variability. More recently, non-linear techniques, including deep domain adaptation and adversarial learning methods, have gained traction in medical image harmonization. These approaches aim to learn domain-invariant representations across imaging sites, often leveraging convolutional neural networks (CNNs) or generative adversarial networks (GANs) (Bashyam et al., 2021; Jeong et al., 2023). While promising, these methods typically require large training datasets and may lack interpretability. A widely used and computationally efficient alternative is NeuroCombat, an extension of the ComBat method originally developed for removing batch effects in genomics data (Johnson et al., 2007), and later adapted for neuroimaging applications (Fortin et al., 2017; Fortin et al., 2018). NeuroCombat relies on regression of covariates to adjust the values of the extracted parameters (e.g., diffusion metrics), creating new parameter maps that are free of scanner-specific effects and comparable across the whole cohort. The expected values are derived using a linear model that incorporates biological variables (e.g., age and sex) alongside additive and multiplicative scanner effects as predictors. To improve the robustness in the estimation of model parameters, Empirical Bayes methods are applied (Fortin et al., 2018; Reynolds et al., 2023). NeuroCombat has shown to increase statistical power compared to other feature-harmonization methods in cross-sectional diffusion metric data (Fortin et al., 2017).

NeuroCombat is a feature-based harmonization method that can be applied to quantitative diffusion metrics obtained from dMRI data. The NeuroCombat approach facilitates the correction of batch effects and enables more accurate analysis of diffusion data across diverse subjects and conditions. To account for longitudinal studies, in which multiple acquisitions of the same subjects are available in different timepoints, LongCombat was created as an extension of NeuroCombat (Beer et al., 2020; Richter et al., 2022). LongCombat aims to estimate and correct for additive and multiplicative scanner effects while accounting for the within-subject correlation inherent to longitudinal studies. Combat-based harmonization methods estimate and remove the site-specific effects using empirical Bayes, which allows borrowing statistical power across sites for better parameter estimation, especially with small sample sizes.

Extensions of the standard ComBat algorithm offer promising potential to enhance the generalization of harmonization techniques applied to diffusion metric maps. To evaluate intra- and inter-scanner variability as well as the effectiveness of harmonization techniques, it is suggested to use a cohort of healthy participants who undergo repeated scans across different scanners. This approach allows for a more reliable assessment of harmonization effects, as well as better generalization of these findings to broader population distributions. By utilizing the same cohort across multiple scanner platforms, researchers can more accurately quantify the impact of harmonization on data consistency and robustness in diverse imaging environments.

The primary objective of this study is to assess the performance of three ComBat-based harmonization methods—NeuroCombat, NeuroCombatScanner and LongCombat—in reducing intra- and inter-scanner variability in diffusion MRI-derived metrics, both at the voxel and region-of-interest (ROI) level. To support this goal, we utilized the B-Q MINDED dataset (Pinto et al., 2025), a recently released inter-scanner test-retest dataset that includes multi-shell diffusion MRI and anatomical T1-weighted images from 38 healthy adult participants. This dataset was specifically designed to enable the evaluation of the comparability of quantitative MRI metrics across scanners and time points. Its test-retest structure, involving acquisitions on multiple scanners, provides an ideal framework for testing harmonization strategies aimed at improving the consistency of MRI-derived measures in multi-site and longitudinal studies.

## 2 Materials and methods

### 2.1 Participants

The B-Q MINDED database consists of anatomical and dMRI scans of 38 highly educated, healthy volunteers aged between 21 and 36 years old (mean ± standard deviation: 27.1 ± 3.4 years). The study cohort was composed of 17 females (mean ± standard deviation: 26.3 ± 3.0 years) and 21 males (mean ± standard deviation: 27.8 ± 3.5 years). All volunteers were scanned between February and December 2020 at the radiology department at the Antwerp University Hospital (UZA) in Antwerp, Belgium. Ethical approval was obtained from the UZA Ethics Committee (B300202042715). Written consent was obtained from all participants.

### 2.2 Image acquisition

The MRI scans were obtained on a 3 Tesla MAGNETOM Skyra and a 3 Tesla MAGNETOM PrismaFit scanner (Siemens Healthineers, Forchheim, Germany). The Siemens Healthineers Skyra scanner had a gradient amplitude of 45 mT/m and slew rate 200 T/m/s and the Siemens Healthineers PrismaFit scanner had a gradient amplitude of 80 mT/m and slew rate 200 T/m/s. The MRI sequences acquired in both scanners included volumetric (1) T1-weighted, (2) multi-shell dMRI optimized for Diffusion Kurtosis Imaging (DKI) and (3) b0 images with inverted phase-encoding.

Base values of the MPRAGE T1-w image (1) were: 0.9 mm isotropic voxels, 192 slices, TR of 2,300 ms, TE of 2.29 ms and TI of 900 ms, 1 average, sagittal orientation and anterior-posterior phase encoding, with acquisition time of 5 min and 21 s. The DKI acquisition (2) had the following acquisition specification: 2.5 mm isotropic voxels, 54 slices, TR of 8,100 ms, TE of 107 ms, single-shot EPI, transversal orientation, posterior-anterior (PA) phase encoding, and 150 diffusion volumes (6 volumes with *b*-value = 0 s/mm^2^, 25 volumes with *b*-value = 700 s/mm^2^, 45 volumes with *b*-value = 1,200 s/mm^2^ and 75 volumes with *b*-value = 2,800 s/mm^2^), with acquisition time of 20 min and 49 s. Additionally, although not used in the current work, six b0 images with inverted phase-encoding, i.e., anterior-posterior (AP), were acquired using the same configuration as the DKI sequence, with an acquisition time of 42 s.

The imaging protocol was acquired twice per subject on both MRI scanners, generating intra-scanner and inter-scanner test and retest data. The first scan for each subject was randomly assigned to PrismaFit or Skyra scanner. The intra-scanner test-retest acquisitions were performed consecutively in the same scanner, with the scanner table being moved out and in of the scanner between the test and retest acquisitions. The time between the MRI acquisitions in the two scanners (PrismaFit and Skyra) was around 1 h. The intra-scanner approach enables the comparison of the scan-rescan variability in subjects who had two scans less than 10 min apart on the same scanner (within-scanner/test-retest cohort, 10 min between the end of the diffusion test scan and the start of the retest scan). The inter-scanner approach allows for the comparison of the scan-rescan variability in subjects who had two scans less than 20 min apart in different scanners (inter-scanner cohort, 20 min between the end of the scan session on the first scanner and the start in the second scanner). In total, our study included 152 diffusion MRI scans from 38 participants (each subject scanned twice on each of the two scanners).

### 2.3 Data availability of the B-Q MINDED dataset

The B-Q MINDED dataset was acquired to investigate scan-rescan variability and inter-scanner effects on diffusion MRI measurements and derived quantitative metrics relevant to brain structural integrity assessment. This dataset, presented and made publicly available for the first time in this manuscript (Zenodo; Pinto et al., 2025),^1^ includes de-identified, BIDS-compliant MRI data from 38 healthy participants, each scanned across test and retest sessions on both Siemens Skyra and PrismaFit scanners. For each subject, the dataset contains defaced T1-weighted anatomical images and associated brain masks, along with pre-processed diffusion-weighted images, including bval, bvec, NIfTI, and JSON files for each diffusion kurtosis imaging (DKI) acquisition. The data is organized by subject and scanner, with DKI files labeled by session (test = 1, retest = 2) and phase-encoding direction (PA: posterior-anterior; AP: anterior-posterior), as indicated in the filenames.

**FIGURE 1:**
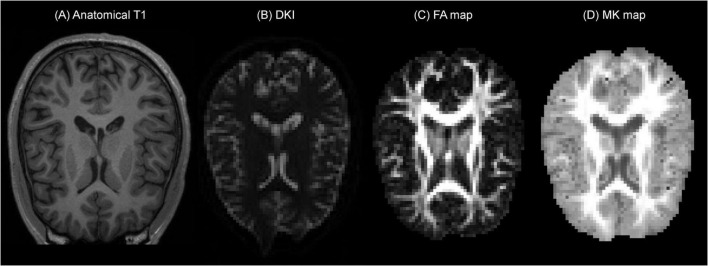
Example of the B-Q MINDED dataset for subject 15 in the PrismaFit scanner acquisition, the extracted **(A)** defaced T1 image on slice 155, **(B)** DKI acquisition with b0 on slice 30, **(C)** the extracted FA map on slice 30, and **(D)** the extracted MK map on slice 30.

A representative example of the B-Q MINDED dataset is depicted in Figure 1, in which the defaced T1 image is shown next to the DKI acquisition and the extracted fractional anisotropy (FA) and mean kurtosis (MK) maps.

### 2.4 Diffusion pre-processing pipeline

**FIGURE 2:**
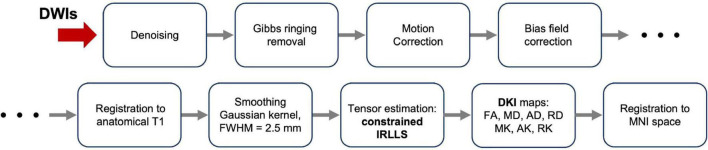
Diagram of processing of the Diffusion Weighted Images (DWIs) from raw images to final diffusion metric maps.

The MRI data were processed using the icobrain diffusion pipeline (Figure 2). Initial preprocessing consisted of corrections for noise using Marchenko-Pastur principal component analysis (MP-PCA) (Veraart et al., 2016), Gibbs ringing (Kellner et al., 2016), head motion and eddy current-induced distortions through affine registration of each diffusion-weighted volume to the first acquired b0 image (Rohde et al., 2004), and scanner bias field (Tustison et al., 2010). Subsequently, susceptibility-induced distortions were corrected through non-linear registration between the diffusion data and the undistorted T1 image (Bhushan et al., 2015; Takatsu et al., 2019). Finally, a Gaussian smoothing filter with a full-width at half-maximum (FWHM) of 2.5 mm (same as the voxel size) was applied prior to model fitting to extract kurtosis maps with reduced noise levels (Tax et al., 2022).

After preprocessing, the diffusion kurtosis estimation was obtained by applying the constrained iterative reweighted linear least squares (constrained IRLLS) fitting procedure (Collier et al., 2015). The combination of Gaussian smoothing and constrained estimation effectively mitigated the issue of “black voxels” in the DKI data, i.e., implausible negative kurtosis estimates typically caused by signal artifacts. Following tensor estimation, the corresponding fractional anisotropy (FA), mean diffusivity (MD), axial diffusivity (AD), radial diffusivity (RD), mean kurtosis (MK), axial kurtosis (AK) and radial kurtosis (RK) maps were derived. Afterward, each individual FA map was non-linearly registered to the FA template of the John Hopkins University (JHU ICBM-DTI-81) atlas. Finally, the transformations were used to project corresponding MD, AD, RD, MK, AK and RK maps to the Montreal Neurologic Institute (MNI) space, a standardized coordinate system in common usage. All registrations (affine and non-rigid) were performed using the open source NiftyReg package (Modat et al., 2014).

### 2.5 Quality control

Although the main imaging artifacts were addressed in the preprocessing steps of the pipeline, targeted quality assessment is key to ensuring reliable results with low measurement errors. Therefore, an automated quality assessment of the available scans was performed based on predefined thresholds, derived from datasets on which the icobrain diffusion software had been previously applied. The following QC metrics were evaluated:

• Brain coverage was assessed to guarantee consistent whole-brain and regional measurements. A standard brain mask in MNI space was transformed into each subject’s native space using an affine transformation. Distances between the bounding box of the transformed mask and the edges of the subject’s FA in native space were computed in six directions (right, left, back, front, down, top). Rejection thresholds were defined as distances less than –10 mm (right, left, back, front), –40 mm (down), and –4.11 mm (top).
• The signal-to-noise ratio (SNR) of the b0 images was used as an indication of the quality of the dMRI data. The SNR was calculated by averaging the b0 images and dividing the resulting signal by the noise map, as extracted from MP-PCA. Finally, the average SNR within a WM mask was computed, with the rejection threshold defined as an SNR less than 10.71.
• The contrast-to-noise ratio (CNR) for each non-zero diffusion shell was calculated using spherical harmonics fit. The angular contrast was computed as the standard deviation of the predicted signal, while noise was defined as the standard deviation of the residuals between the actual and predicted signals. The resulting CNR map was then averaged within a WM mask, with the rejection threshold defined as a CNR less than 5.06.
• The distribution of the diffusion gradient directions was assessed to evaluate the uniformity of sampling using built-in functions available in the MRtrix3 software framework (Tournier et al., 2019).
• Motion was quantified as the average root-mean-square (RMS) displacement introduced by the series of affine transformations used to align each volume with the first acquired b0 image (reference image). The rejection threshold was set at 15 mm.
• The goodness of alignment to the structural T1 was quantified using normalized mutual information, with a lower bound of 0.095.
• Finally, the IRLLS estimation technique produces a binary outlier map where data points classified as intensity outliers are labeled as 1, and accepted data points as 0. This output was used to provide a shell-wise overview of the outlier distribution per slice (axial, coronal, and sagittal) and per volume, supporting the final QC decision.

All available scans successfully passed the automated quality assessment. In addition, manual QC was performed by the primary and secondary authors to assess the quality of the original diffusion and anatomical data, as well as the registration to MNI space. No issues were identified.

### 2.6 White matter masks

**FIGURE 3:**
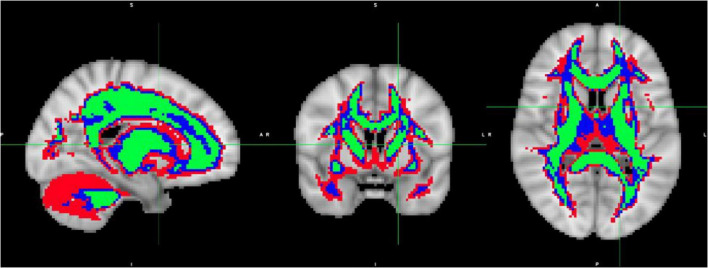
White Matter masks created based on the average over the subject’s diffusion FA maps registered to MNI space: WMmask01 with FA threshold of 0.1 (depicted in red), WMmask02 with FA threshold 0.2 (in blue) and WMmask03 with FA threshold 0.3 (in green).

To analyze the diffusion maps, specific regions of interest (ROIs) were identified, according to the brain microstructure. Image masks were created to contain the white matter brain region. The white matter masks were created based on the average of the subjects’ diffusion FA maps registered to MNI space, considering the FA threshold to identify the WM of the brain. Three (3) thresholds were used to create the following WM masks: WMmask01 with FA threshold of 0.1, WMmask02 with FA threshold of 0.2 and WMmask03 with FA threshold of 0.3 (depicted in green on Figure 3).

### 2.7 Harmonization approaches: NeuroCombat and LongCombat

**FIGURE 4:**
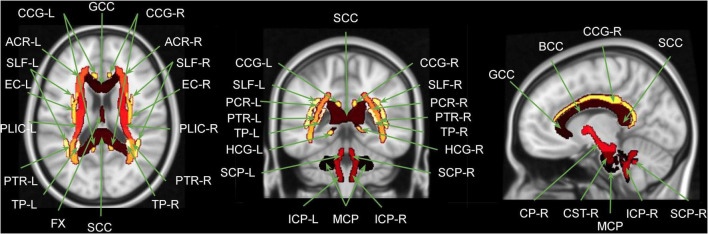
Overlap between MNI brain template and the JHU WM atlas regions. GCC, genu corpus callosum; BCC, body corpus callosum; SCC, splenium corpus callosum; FX, fornix; MCP, middle cerebellar peduncle; [R/L], right and left; CP, cerebral peduncle; ICP, inferior cerebellar peduncle; SCP, superior cerebellar peduncle; CST, corticospinal tract; ACR, anterior corona radiate; PCR, posterior corona radiate; SLF, superior longitudinal fasciculus; EC, external capsule; PLIC, posterior limb of internal capsule; CCG, cingulate gyrus part of the cingulum; HCG, hippocampus part of the cingulum; PTR, posterior thalamic radiation; TP, tapetum. Figure adapted from Siqueira Pinto et al. (2023).

Harmonization was performed using the R (version 4.3.2) packages: neuroCombat version 1.0.13 (Fortin et al., 2017, 2018)^2^ and longCombat version 0.0.0.90000 (Beer et al., 2020).^3^ Harmonization was performed separately for each of the diffusion metric maps (FA, MD, AD, RD, MK, AK, and RK) on a voxel-wise level and on a ROI-wise level using NeuroCombat and LongCombat. For the voxel-wise harmonization, the voxel intensities of the diffusion metric maps within WMmask01 (total of 64,667 voxels) were used as the input values in the harmonization algorithms. For the ROI-wise harmonization, the average of the voxel intensities of the diffusion metric maps within the specific ROIs were used as the input values in the harmonization algorithms. The 51 specific ROIs used were WMmask01, WMmask02, WMmask03 (see Figure 3) and the 48 JHU regions (see Figure 4). In this study, age and sex served as biological covariates while fitting the harmonization models.

The diffusion metric maps were harmonized in six manners: (1) NeuroCombat voxel: NeuroCombat voxel-wise with 4 scanners/batches to be corrected for: PrismaFit test, PrismaFit retest, Skyra test and Skyra retest, (2) NeuroCombat ROI: NeuroCombat ROI-wise with 4 scanners to be corrected for: PrismaFit test, PrismaFit retest, Skyra test and Skyra retest, (3) NeuroCombatScanner voxel: NeuroCombat voxel-wise with 2 scanners to be corrected for: PrismaFit and Skyra, all test/retest data was used while implementing subject ID as an additional biological covariates, which we will call NeuroCombatScanner voxel-wise, (4) NeuroCombatScanner ROI: NeuroCombat ROI-wise with 2 scanners to be corrected for: PrismaFit and Skyra, all test/retest data was used while implementing subject ID as an additional biological covariates, which we will call NeuroCombatScanner ROI-wise, (5) LongCombat voxel: LongCombat voxel-wise with 2 scanners to be corrected for: PrismaFit and Skyra, 2 timepoints to be corrected for: test and retest and a subject-specific random intercept, and (6) LongCombat ROI: LongCombat ROI-wise with 2 scanners to be corrected for: PrismaFit and Skyra, 2 timepoints to be corrected for: test and retest and a subject-specific random intercept.

ComBat-based harmonization methods are designed to adjust for site-specific variability in multi-site neuroimaging data by estimating and removing scanner-related effects through an empirical Bayes framework. These methods model the unwanted variation as a combination of additive and multiplicative site effects, which are then statistically adjusted to align the data across scanners or sites. The empirical Bayes approach enables the method to “borrow statistical power” across all participating sites, effectively pooling information to improve the estimation of these site-specific parameters—particularly beneficial when some sites have small sample sizes (Fortin et al., 2017, 2018). This approach helps ensure that the harmonized data retain biologically meaningful signals, such as effects of age, sex, or clinical diagnosis, while reducing spurious differences that are solely due to acquisition conditions.

### 2.8 Statistical analysis

All statistical analysis was performed in R (version 4.3.2) (R Core Team, 2021). The statistical analysis was performed in the 51 chosen ROIs (WMmask01, WMmask02, WMmask03 and the 48 JHU regions) for the original diffusion MRI maps (FA, MD, AD, RD, MK, AK, and RK), and on the maps harmonized according to the six methods mentioned above.

The original non-harmonized diffusion data metric maps (FA, MD, AD, RD, MK, AK, and RK) were tested for normality considering the mean intensity value within the WMmask01 of all 38 subjects of the B-Q MINDED subdatasets (PrismaFit test, PrismaFit retest, Skyra test and Skyra retest). Each time, the normality of the 38-sized dataset was checked by visual inspection via Q-Q plot (Dodge, 2008) and the Shapiro-Wilk test (Shapiro and Wilk, 1965), which rejects the hypothesis of normality when the *p*-value is less than or equal to 0.05.

Next, paired *t*-tests were used to compare two population means and standard deviations (of diffusion metric intensities within ROIs) for two samples that are correlated, to determine if there is a significant difference between the two groups (intra- or inter-scanner populations). The *t*-test result was considered statistically significant if *p* < 0.05.

To further evaluate the effects of harmonization on measurement reproducibility and biological variability, we computed three complementary metrics: the within-subject coefficient of variation (CVws) (Quan and Shih, 1996), the between-subject coefficient of variation (CVbs), and the intraclass correlation coefficient (ICC) (McGraw and Wong, 1996). These metrics were calculated for each diffusion metric in the WM regions of interest, separately for intra-scanner and inter-scanner comparisons, both before and after harmonization.

CVws was calculated as the standard deviation of the test-retest differences within each subject, divided by the subject’s mean across sessions, and then averaged across subjects. CVbs was computed as the standard deviation of the subject-level means across the cohort, divided by the overall mean, to estimate biological variability. The ICC was used to assess the reliability of individual differences by quantifying the proportion of total variance attributable to between-subject variability. Specifically, we employed the ICC(A,1) model—based on a two-way mixed-effects framework with absolute agreement of single measurements (McGraw and Wong, 1996). All calculations were implemented in R, using the psych package for ICC estimation and custom scripts for CVws and CVbs computations. These analyses quantify how harmonization affected measurement error, biological signal preservation, and subject-level reliability across scanners and sessions.

Lastly, an analysis of variance (ANOVA) was performed to compare the groups (PrismaFit test, PrismaFit retest, Skyra test and Skyra retest). For the dMRI measures, two-way ANOVA was performed to evaluate the effect of scanner, test-retest, the interaction between scanner and test-retest and subject ID. The comparison was performed for all masks and JHU regions. Test results with *p* < 0.05 were considered statistically significant. As this was an exploratory analysis, no corrections for multiple comparisons were performed.


(1) D⁢i⁢f⁢f⁢u⁢s⁢i⁢o⁢n⁢M⁢a⁢p∼T⁢e⁢s⁢t⁢R⁢e⁢t⁢e⁢s⁢t*S⁢c⁢a⁢n⁢n⁢e⁢r+S⁢u⁢b⁢j⁢e⁢c⁢t⁢I⁢D

The diffusion maps (FA, MD, AD, RD, MK, AK, and RK) were evaluated by the two-way ANOVA test following the R implementation based on equation 1, in which *Diffusion Map* is the mean intensity of the diffusion metric over the ROI voxels, for each of the diffusion maps separately, *TestRetest* is the intra-scanner factor, *Scanner* is the inter-scanner factor and *Subject ID* is the individual’s identification as each subject obtained 4 sets of diffusion maps (PrismaFit test, PrismaFit retest, Skyra test and Skyra retest). Thus, the individual’s ID, the intra-scanner effect, the inter-scanner effect and the interaction between both can be evaluated by the two-way ANOVA test.

Linear mixed-effects (lme) models extend simple linear models by incorporating both fixed effects (effects that are consistent and repeatable across different groups) and random effects (effects that vary intra- and inter-scanner). In R, the lme4 package was used to provide robust functions to fit lme models. In this study, age and sex were considered fixed effects, while scanner was considered random effects. The lme model was implemented on the diffusion data before and after harmonization to evaluate how the biological information (age and sex) was maintained after harmonization.


(2) D⁢i⁢f⁢f⁢u⁢s⁢i⁢o⁢n⁢M⁢a⁢p∼A⁢g⁢e*S⁢e⁢x+S⁢c⁢a⁢n⁢n⁢e⁢r

The diffusion maps (FA, MD, AD, RD, MK, AK, and RK) were used in the linear mixed-effect model as described by equation 2. The lme model considers the effect of *Scanner*, *Age* and *Sex* and the interaction of the last two, and how it relates to the mean intensity of each Diffusion Map.

## 3 Results

The diffusion data was tested for normality, both visually via the QQ plot, and statistically, via the Shapiro-Wilk’s test. Based on the results of these normality tests, the non-harmonized diffusion metrics averaged within the WMmask01 (FA, MD, AD, RD, MK, AK, and RK) were found to exhibit a normal distribution within the B-Q MINDED dataset.

**FIGURE 5:**
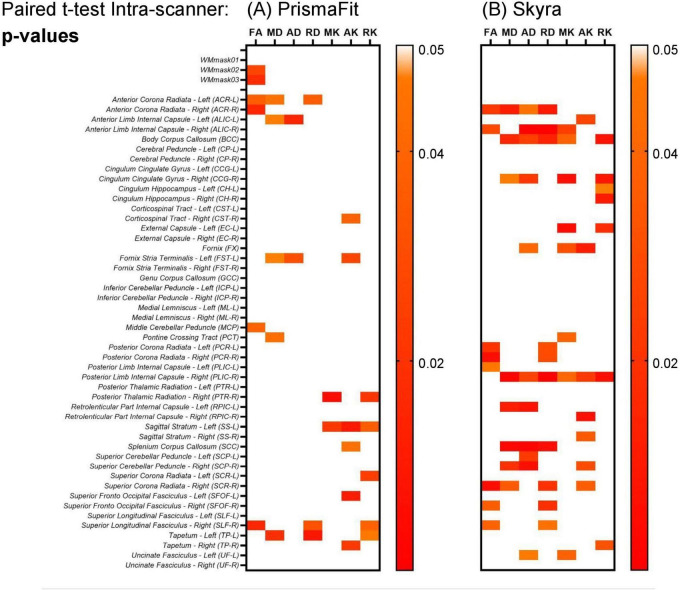
Heatmaps for the *p*-values obtained from the paired t-test on the non-harmonized diffusion metric maps on the evaluation intra-scanner for the test and retest data obtained on the PrismaFit **(A)** and Skyra **(B)** scanners: FA, MD, AD, RD, MK, AK, and RK.

A paired *t*-test was performed on the original non-harmonized data to evaluate the presence of intra-scanner differences in the diffusion metric maps (FA, MD, AD, RD, MK, AK, and RK) for each of the Siemens Healthineers scanners (PrismaFit and Skyra). The results are shown in Figure 5. The results show that for the PrismaFit scanner, there are a few significant (*p* < 0.05) intra-scanner differences in specific WM regions for multiple diffusion metric maps. Indeed in 16 out of the 51 regions, at least one diffusion map presents a significant intra-scanner variation. For the Skyra scanner, significant (*p* < 0.05) intra-scanner differences are observed: in 21 out of the 51 WM regions, at least one diffusion map presented a significant variation.

**FIGURE 6:**
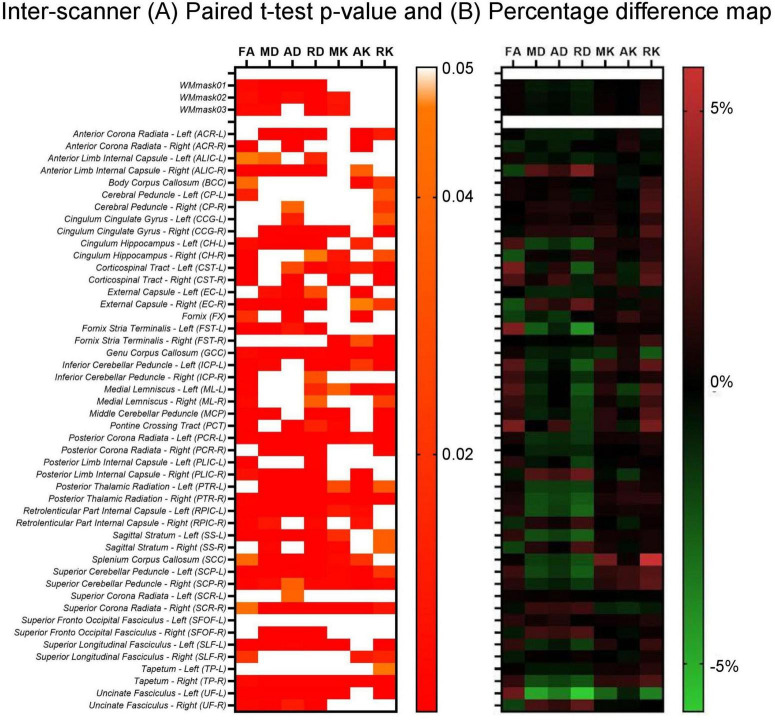
Inter-scanner heatmaps between Skyra test vs. PrismaFit test for the **(A)** *p*-values obtained from the paired t-test on the non-harmonized diffusion metrics obtained on the PrismaFit and Skyra scanners: FA, MD, AD, RD, MK, AK, and RK; and **(B)** percentage difference map between the PrismaFit and Skyra scanners.

In addition, the paired *t*-test was also performed to evaluate the inter-scanner differences in the non-harmonized diffusion metrics for the specific WM regions. The results are shown in Figure 6. It can be observed in Figure 6A, which shows the paired *t*-test results, that for all original (non-harmonized) diffusion metrics there are multiple WM regions in which the scanner effect is significant (*p* < 0.05), while 7/51 WM regions have significant scanner effects for all diffusion metrics. Additionally, as can be observed in Figure 6B, in which the percentage inter-scanner difference is depicted, the larger WM ROIs show only small differences (< 1%) and there is not a clear pattern in the inter-scanner variation of the diffusion values.

**FIGURE 7:**
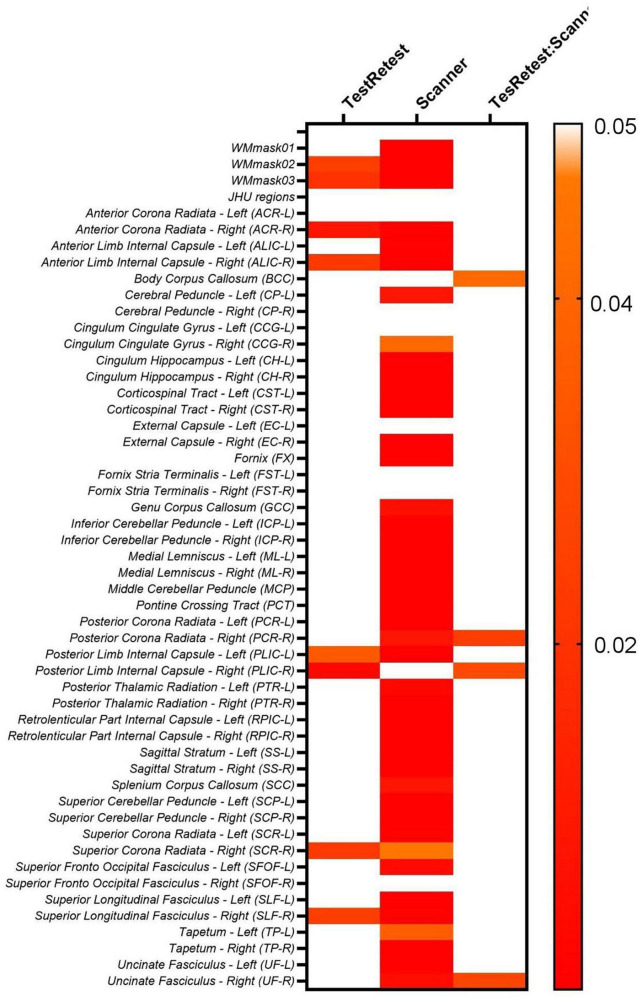
Heatmaps for the *p*-values obtained from the two-way ANOVA test on the original FA maps for both PrismaFit and Skyra scanners including test and retest data.

Additionally, the two-way ANOVA test was performed to also evaluate the intra- and inter-scanner variability of the 2 scanners used in the study on the non-harmonized diffusion metrics (FA, MD, AD, RD, MK, AK, and RK), Figure 7 displays the results for FA.

The results shown in Figure 7 demonstrate that for the original non-harmonized FA maps, 8/51 of the WM regions considered display significant intra-scanner (TestRetest factor in Equation 1) effects, namely WMmask02, WMmask03, ACR-R, ALIC-R, PLIC-L, PLIC-R, SCR-R and SLF-R. Moreover, 44/51 WM regions indicate significant inter-scanner effects (Scanner factor in Equation 1). For the interaction between intra- and inter-scanner effect, 4/51 regions showed significant effects, namely BCC, PCR-R, PLIC-R and UF-R. Additionally, it was not added in the table, but subject ID was always significant for this analysis for all diffusion maps, for all WM regions.

When evaluating the other diffusion maps, similar patterns were identified. For MD, 34/51 WM regions showed significant inter-scanner effects, while 5/51 regions are significant for intra-scanner effects, namely PLIC-R, SCC, CCG-R, TP-L, and SFOF-R, and 1/51 WM regions showed a significant intra-inter-scanner effect, namely ALIC-R. AD demonstrated significant intra-scanner effects for 3/51 WM regions, namely for SCC, TP-L, and FX, and significant inter-scanner and interaction between intra- and inter-scanner for ALIC-R. RD displayed significant intra-scanner effects for 9/51 WM regions, namely ALIC-R, PLIC-R, SCC, TP-L, PCR-L, ACR-R, SCR-R, SLF-R, and SFOF-R, and significant inter-scanner and interaction between intra- and inter-scanner for ALIC-R.

For the kurtosis parameters, a similar pattern is observed. For the non-harmonized MK maps, 38/51 WM regions are significant for inter-scanner effect, 3/51 are significant for intra-scanner effects, as PTR-R, EC-L, and CCG-R, while CCG-R and PLIC-R displays significant interaction between intra- and inter-scanner effect. For AK, the FX is the only WM region with significant intra-scanner effect, while TP-R, SCP-R, and ALIC-L are identified to have significant interaction between intra- and inter-scanner effects. For RK maps, CCG-R is the only WM region with significant intra-scanner effect, and significant interaction between intra- and inter-scanner effects were found for PLIC-R and CCG-R.

**FIGURE 8:**
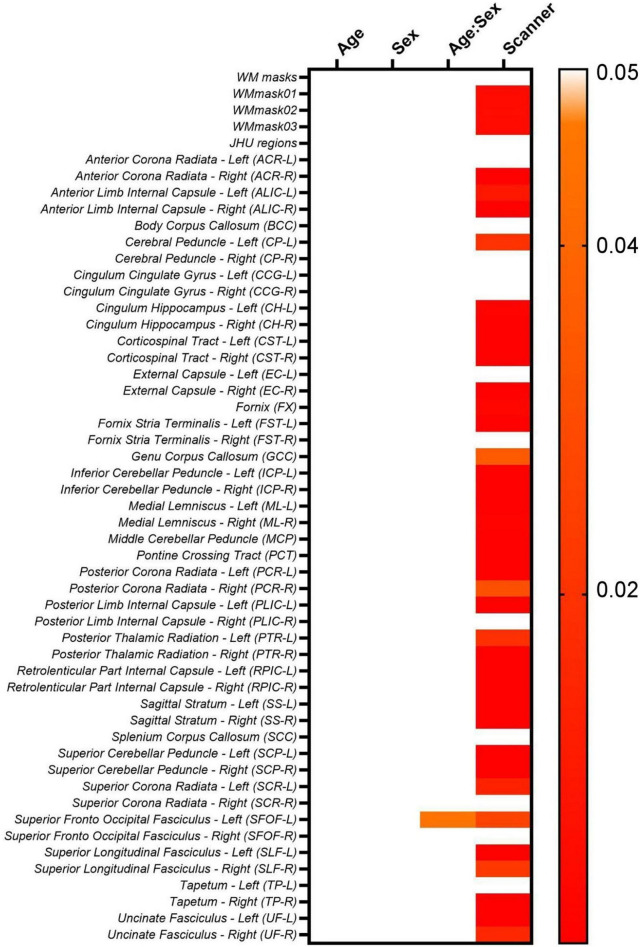
Heatmaps for the *p*-values obtained from the lme model on the original non-harmonized FA maps.

Moreover, to investigate if the biological (age and sex) effects are maintained while intra- and inter-scanner factors are removed during the data harmonization, we first check if the biological effects are statistically significant in the non-harmonized diffusion metric maps via lme (Equation 2). The results for the FA maps are shown in Figure 8. It follows from Figure 8 that for the FA maps age and sex are not statistically significant for none of the WM regions, however, the interaction between age and sex is significant for the SFOF-L. Additionally, most WM regions have statistically significant scanner effects. The scanner effects were found to be similar for all diffusion metric maps evaluated.

However, for MD, age is significant statistically for EC-R and PLIC-R, while sex is significant for EC-R and SCR-L. For AD, only PLIC-R has significant relation to age. RD shows significant relation to age for EC-R and CCG-L. Looking at the relations for MK, age is significant for PCT. For AK, age is significantly correlated for PLIC-R, sex is significantly correlated to PLIC-R, SSL, and CCG-R, and the age-sex interaction is also significantly correlated to PLIC-R, SSL, and CCG-R. And for RK, no WM regions are significantly related to the biological effects.

Based on the results presented thus far, which pertain to non-harmonized intra- and inter-scanner data, it is evident that data harmonization is necessary to mitigate scanner- and acquisition-specific effects. This is crucial for accurately assessing the biological parameters associated with the diffusion metrics. Thus, below we describe the results obtained with the six harmonization approaches described in the methods section: (1) NeuroCombat voxel, (2) NeuroCombat ROI, (3) NeuroCombatScanner voxel, (4) NeuroCombatScanner ROI, (5) LongCombat voxel, and (6) LongCombat ROI.

**FIGURE 9:**
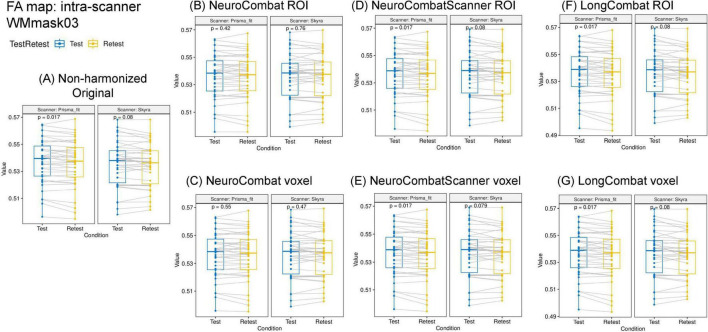
Data distribution of mean FA values within the WMmask03 for intra-scanner comparisons before and after harmonization for the PrismaFit and Skyra scanners: **(A)** Non-harmonized - Original, **(B)** NeuroCombat ROI, **(C)** NeuroCombat voxel, **(D)** NeuroCombatScanner ROI, **(E)** NeuroCombatScanner voxel, **(F)** LongCombat ROI, and **(G)** LongCombat voxel. The paired *t*-test *p*-value for each of the intra-scanner comparisons is shown in the graphs.

The six harmonization algorithms were implemented for the diffusion metric maps (FA, MD, AD, RD, MK, AK, and RK) and the impact of the harmonization on the intra- and inter-scanner differences was evaluated. Figure 9 shows the results of the comparison of intra-scanner differences before and after harmonization for the FA map within the WMmask03. Similar patterns were identified for the other diffusion metric maps and for the additional WM regions.

Next to the distribution of the mean FA within the WMmask03 before and after harmonization, Figure 9 also shows the *p*-values of the paired *t*-test that was implemented to test intra-scanner differences before and after harmonization. Results are shown for all six harmonization approaches listed above. The non-harmonized data demonstrated a significant (*p* < 0.05) intra-scanner relation for PrismaFit with a *p*-value of 0.017 and non-significant *p*-value of 0.08 for Skyra. After the harmonization via NeuroCombat ROI- and voxel-wise, the test-retest effects are removed and the intra-scanner relations turn non-significant, both for PrismaFit (*p*-value of 0.42 and 0.55, respectively), and Skyra (*p*-value of 0.76 and 0.47, respectively). For the other methods, NeuroCombatScanner and LongComBat ROI- and voxel-wise, the level of intra-scanner significance remained the same as for the original data.

**FIGURE 10:**
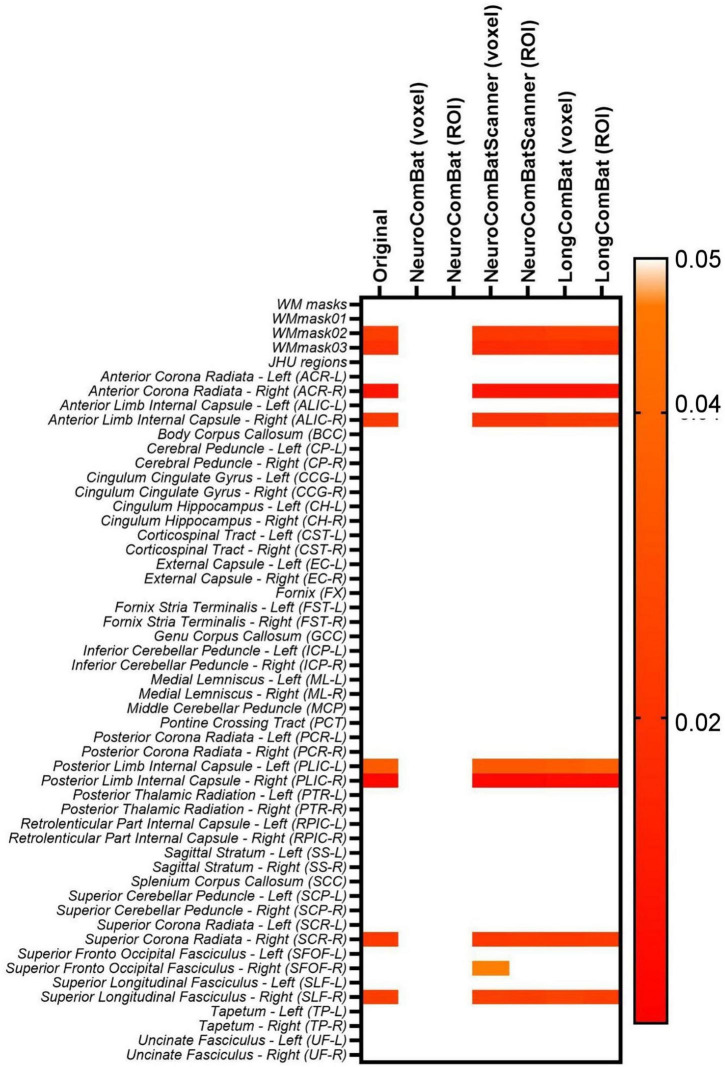
Heatmap for the *p*-values obtained from the two-way ANOVA test for the intra-scanner (TestRetest) variable on the original non-harmonized FA maps and in the harmonized maps via NeuroCombat voxel, NeuroCombat ROI, NeuroCombatScanner voxel, NeuroCombatScanner ROI, LongCombat voxel, and LongCombat ROI.

Additionally, the two-way ANOVA test was performed pre- and post-harmonization of the diffusion metric maps; the results for the WM regions for the FA maps are shown in Figure 10. The results demonstrate the same pattern as identified in the paired *t*-test, as expected. While NeuroCombat voxel and ROI were able to correct for the significant (*p* < 0.05) intra-scanner effects in specific WM regions (WMmask02, WMmask03, ACR-R, ALIC-R, PLIC-R, PLIC-L, SCR-R and SLF-R), the other harmonization methods (NeuroCombat Scanner and LongCombat) did not remove the significant intra-scanner differences. Similar patterns were identified for the other diffusion metric maps.

**FIGURE 11:**
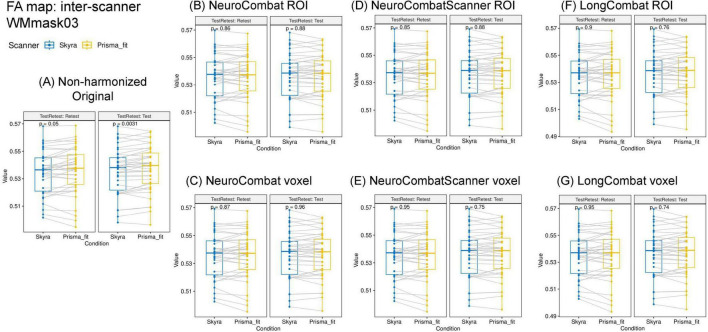
Distribution of the mean FA values within the WMmask03 for inter-scanner comparisons before and after harmonization: **(A)** Non-harmonized—Original, **(B)** NeuroCombat ROI, **(C)** NeuroCombat voxel, **(D)** NeuroCombatScanner ROI, **(E)** NeuroCombatScanner voxel, **(F)** LongCombat ROI, and **(G)** LongCombat voxel. The paired *t*-test *p*-value for each of the intra-scanner comparisons is shown in the graphs.

Furthermore, we evaluated if the harmonization approaches were able to remove the scanner effect, which for non-harmonized data was observed to be very significant for multiple WM regions (Figure 6). The six harmonization algorithms were implemented for the diffusion metric maps (FA, MD, AD, RD, MK, AK, and RK) and the impact of the harmonization on the inter-scanner differences was evaluated. Figure 11 shows the results of the comparison of inter-scanner differences before and after harmonization for the FA map within the WMmask03.

The results of the paired *t*-test for inter-scanner evaluation are displayed for the FA map of the WMmask03 in Figure 11. Similar patterns were observed across the other diffusion metrics and WMmask regions, where all harmonization methods effectively eliminated scanner-specific effects while preserving the underlying data distribution.

**FIGURE 12:**
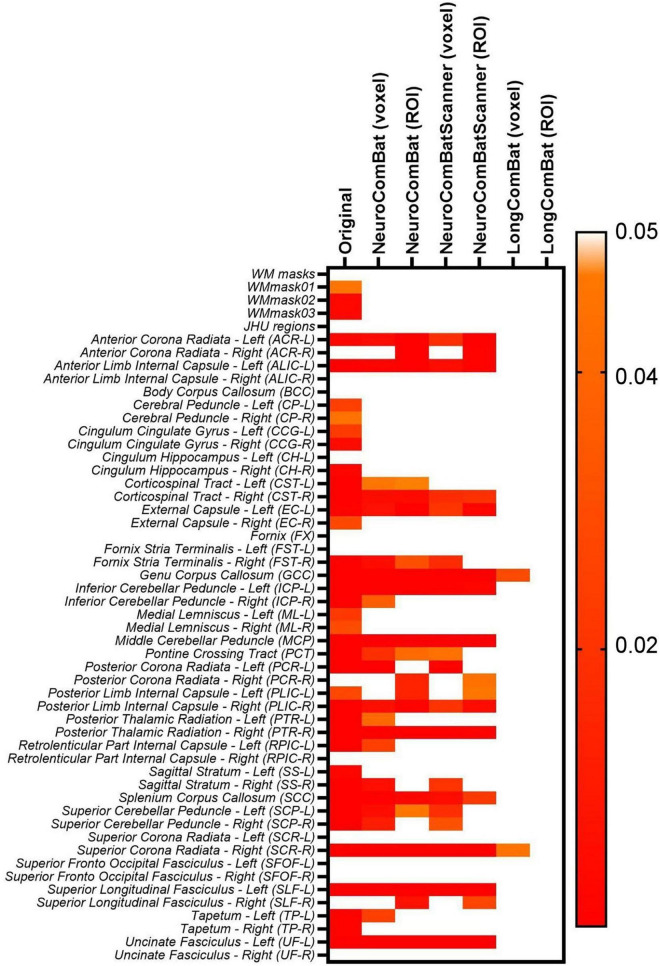
Heatmap for the *p*-values obtained from the two-way ANOVA test for the inter-scanner (Scanner) variable on the original non-harmonized MK maps and in the harmonized maps via NeuroCombat voxel, NeuroCombat ROI, NeuroCombatScanner voxel, NeuroCombatScanner ROI, LongCombat voxel, and LongCombat ROI.

However, when looking at the smaller WM regions from the JHU atlas, it was possible to identify that for various regions there was an optimal harmonization approach that more effectively removed the scanner effects than other approaches. Indeed, the LongCombat voxel and ROI were the harmonization approaches that increased the *p*-value for the paired *t*-test and two-way ANOVA consistently for all evaluated regions, removing all significant scanner effects on the diffusion data. The results on Figure 12 for the two-way ANOVA evaluation for the MK data demonstrate that clearly. A very similar pattern was identified for the other diffusion metric maps, in which LongCombat again performed best when it comes to the removal of scanner effects.

**FIGURE 13:**
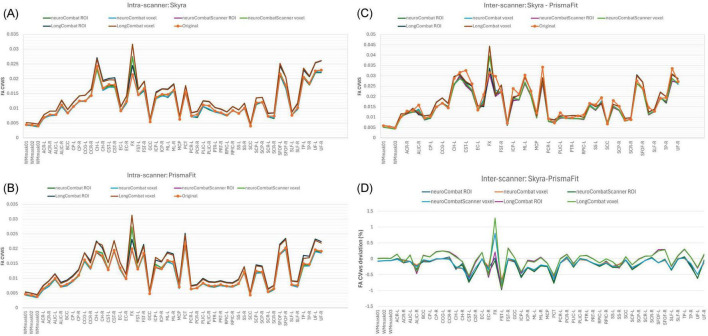
Within-subject coefficient of variation (CVws) for FA maps before and after harmonization. **(A)** CVws intensity values across the original and harmonized data (NeuroCombat, NeuroCombatScanner, and LongCombat), computed both ROI- and voxel-wise, for the Skyra intra-scanner comparison (Skyra test vs. Skyra retest); **(B)** for the PrismaFit intra-scanner comparison (PrismaFit test vs. PrismaFit retest); **(C)** for the Skyra-PrismaFit inter-scanner comparison (Skyra test vs. PrismaFit test) and **(D)** change in CVws for the inter-scanner comparison (Skyra test vs. PrismaFit test).

**FIGURE 14:**
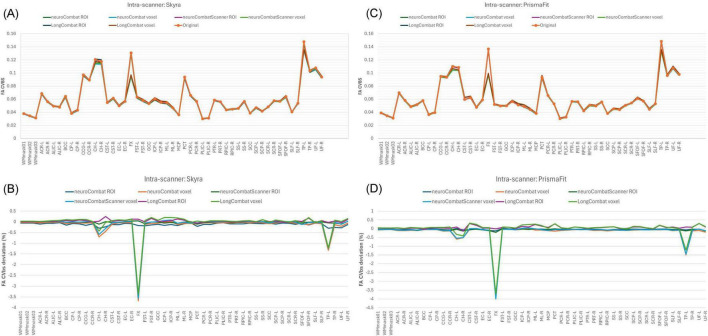
Between-subject coefficient of variation (CVbs) for FA maps before and after harmonization. **(A)** CVbs intensity values across the original and harmonized data (NeuroCombat, NeuroCombatScanner, and LongCombat), computed both ROI- and voxel-wise, for the Skyra intra-scanner comparison (Skyra test vs. Skyra retest); **(B)** change in CVbs (harmonized—original) for the Skyra intra-scanner comparison; **(C)** CVbs intensity values across the original and harmonized data for the PrismaFit intra-scanner comparison (PrismaFit test vs. PrismaFit retest) and **(D)** change in CVbs (harmonized—original) for the PrismaFit intra-scanner comparison.

**FIGURE 15:**
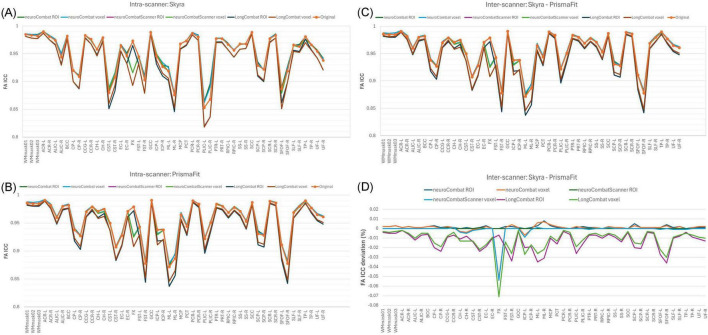
Intraclass Correlation Coefficient (ICC) for FA maps before and after harmonization. **(A)** ICC intensity values across the original and harmonized data (NeuroCombat, NeuroCombatScanner, and LongCombat), computed both ROI- and voxel-wise, for the Skyra intra-scanner comparison (Skyra test vs. Skyra retest); **(B)** for the PrismaFit intra-scanner comparison (PrismaFit test vs. PrismaFit retest); **(C)** for the Skyra-PrismaFit inter-scanner comparison (Skyra test vs. PrismaFit test) and **(D)** Change in ICC for the inter-scanner comparison (Skyra test vs. PrismaFit test).

To evaluate the impact of harmonization on measurement consistency and biological variability, we calculated the within-subject coefficient of variation (CVws), between-subject coefficient of variation (CVbs), and intraclass correlation coefficient (ICC) for each diffusion metric across all WM regions, both before and after harmonization. Results for FA are presented in Figures 13–15. Similar trends were observed across other diffusion metrics (MD, AD, RD, MK, AK, and RK), and we describe the main findings below based on the FA results.

In the intra-scanner CVws assessment for FA (Figures 13a,b), both NeuroCombat and NeuroCombatScanner, applied ROI- and voxel-wise, showed either reduced or minimally altered CVws compared to the original data for both Skyra and PrismaFit scans. In contrast, LongCombat implementations, surprisingly, demonstrated positive differences from the original CVws values. Across all harmonization methods, the largest increases in CVws from the original data were observed in the fornix (FX), which may reflect the region’s heightened sensitivity to partial volume effects, motion artifacts, or its small size and anatomical proximity to cerebrospinal fluid spaces.

For the inter-scanner CVws comparison (Skyra test vs. PrismaFit test; Figures 13C,D), all harmonization methods resulted in relatively minor changes from the original CVws values. NeuroCombat tended to decrease CVws slightly, NeuroCombatScanner remained closest to the original measurements, and LongCombat showed an overall increase in CVws, suggesting a potential overcorrection in the inter-scanner context.

In the intra-scanner assessment of between-subject variability (CVbs) for FA (Figure 14), all harmonization methods—NeuroCombat, NeuroCombatScanner, and LongCombat—applied both ROI- and voxel-wise, resulted in only minimal changes compared to the original data for both the Skyra and PrismaFit scans. The CVbs patterns were highly consistent across the two scanners, suggesting that the harmonization procedures preserved overall biological variability between subjects in a reproducible manner. However, among the voxel-wise harmonization approaches, the largest deviations from the original CVbs values were observed in the fornix (FX), with smaller deviations noted in the left tapetum (TP-L) and the left cingulum hippocampus (CH-L), indicating some regional sensitivity to harmonization effects.

In the intra-scanner assessment of intraclass correlation coefficients (ICC) for FA (Figures 15a,b), both NeuroCombat and NeuroCombatScanner—applied at the ROI and voxel levels—resulted in ICC values that were either slightly reduced or largely unchanged compared to the original data for both the Skyra and PrismaFit scans. In contrast, LongCombat (both ROI- and voxel-wise) led to more pronounced decreases in ICC, indicating reduced test-retest reliability following harmonization. The largest deviations in ICC were observed in the fornix (FX) for both NeuroCombat and LongCombat when applied voxel-wise, with smaller differences appearing in other white matter regions.

A similar pattern was observed in the inter-scanner ICC comparison (Skyra test vs. PrismaFit test; Figures 15C,D). Again, NeuroCombat and NeuroCombatScanner produced minimal changes in ICC values, while LongCombat consistently resulted in greater reductions. The fornix (FX) remained the region with the largest ICC decrease, particularly in voxel-wise analyses, underscoring its heightened sensitivity to harmonization effects.

**FIGURE 16:**
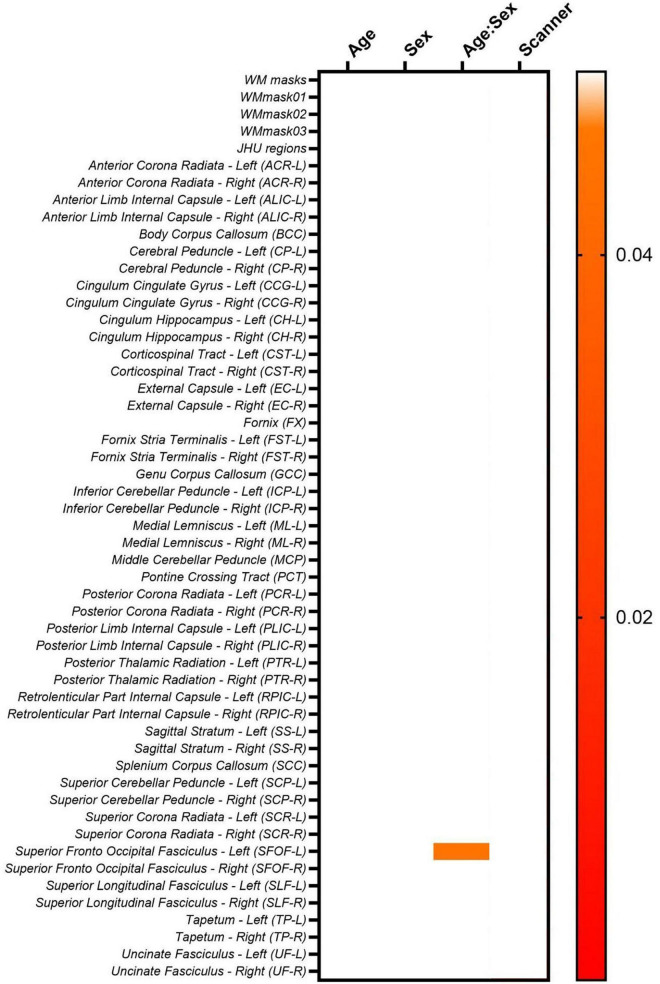
Heatmap for the *p*-values obtained from the lme model on the LongCombat ROI FA maps.

Lastly, Figure 16 shows the lme results for the LongCombat ROI harmonized FA maps. It is observed that age and sex are not statistically significant for any of the WM regions, however, the interaction between age and sex is significant for SFOF-L, as similarly demonstrated in the non-harmonized original data (Figure 8). Additionally, all scanner effects are removed. The corrections of the scanner effects are similar for all diffusion metric maps evaluated while keeping the interactions with age, sex and the interaction between age and sex.

The biological (age and sex) effects on the diffusion data were evaluated via a lme after data harmonization (Equation 2). Unfortunately, for this dataset the biological effects considered do not have a significant relation to the diffusion metric maps, before nor after harmonization. The lme evaluation demonstrates the same pattern identified previously, in which LongCombat voxel and ROI were the best approaches to remove significant scanner effects while seemingly preserving the overall underlying relation with the biological features.

**FIGURE 17:**
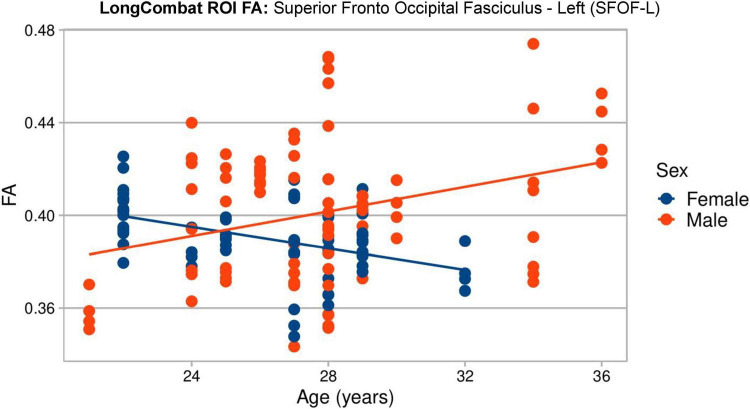
Graphical visualization of all LongCombat ROI FA intensities for Superior Fronto Occipital Fasciculus - Left (SFOF) across the subjects ages, color coded by sex (male in red and female in blue).

A visual representation of the significant age:sex relation in the SFOF-L FA LongCombat ROI data is shown in Figure 17. In the figure it is possible to identify different trends for the FA data with age for the two biological sexes, with a positive trend with age for males and a negative trend for females. The individual evaluations for age and sex separately is not significant, however, the interaction between both biological effects provides a significant relation on the non-harmonized data and such is maintained after LongCombat harmonization, for both ROI and voxel implementations.

## 4 Discussion

This study presented a comprehensive evaluation of inter- and intra-scanner ComBat-based harmonization methods using neuroimaging data from the B-Q MINDED dataset, in which the same subjects were scanned multiple times across multiple scanners. We demonstrated an overall robust performance of NeuroCombat and LongCombat in harmonizing cross-sectional and longitudinal diffusion metrics, both at the voxel and region-of-interest (ROI) levels. However, we note that multiple comparisons were performed across a large number of ROIs and diffusion metrics without formal correction procedures (e.g., FDR or Bonferroni). While this approach is appropriate in the context of exploratory analysis, we advise caution in interpreting isolated significant findings, as they may be susceptible to false positives. Future studies with larger samples and more targeted hypotheses would benefit from applying statistical corrections to more rigorously assess robustness.

Starting with the original non-harmonized diffusion metric maps (FA, MD, AD, RD, MK, AK, and RK), this study demonstrated that even for scanners of the same brand, located in the same hospital and being operated by the same personnel, there is significant intra- and inter-scanner variability when evaluating the extracted metric maps (see Figures 5, 6).

Interestingly, notable variations were observed in the intra-scanner evaluations for the two Siemens Healthineers scanners that were assessed (3T PrismaFit and 3T Skyra). These discrepancies suggest that each scanner may exhibit unique characteristics or performance profiles, which could influence the consistency and reliability of the data obtained. Such differences may be attributed to factors such as hardware specifications, calibration procedures, or even the specific scanning protocols employed by each device. Understanding and accounting for these intra-scanner variations is essential for ensuring accurate data interpretation and minimizing potential sources of error in scientific studies that rely on scanning technology.

In more detail, for the PrismaFit scanner, the number of WM regions in which statistically significant (*p* < 0.05) intra-scanner differences for at least one diffusion map were found was 16 out of 51, while for the Skyra scanner this number increased to 21 out of 51 (Figure 5). For the PrismaFit scanner, the following WM regions showed significant intra-scanner variation in at least three diffusion maps: Anterior Corona Radiata - Left (ACR-L), Superior Longitudinal Fasciculus - Right (SLF-R) and Tapetum - Left (TP-L). For the Skyra scanner, the following WM regions showed significant intra-scanner variation in at least five diffusion maps: Posterior Limb Internal Capsule - Right (PLIC-R), Anterior Limb Internal Capsule - Right (ALIC-R) and Body of Corpus Callosum (BCC). The inter-scanner differences between the PrismaFit and Skyra could be caused by differences in gradient performance and different bore size, which could cause (minor) differences in field homogeneity and can be considered a factor in the inter-scanner evaluations.

Additionally, the results of the paired *t*-test that was applied to the original, non-harmonized diffusion metric maps obtained from the PrismaFit test data and Skyra test data demonstrate that for several WM regions the scanner effect is significant (*p* < 0.05). In fact, 7 out of 51 WM regions present significant scanner effects for all diffusion metric maps, namely GCC, PCR-L, PTR-R, SCP-L, SCP-R, SCR-R, and TP-R. When looking at the inter-scanner percentage difference map shown in Figure 6B, larger WM structures demonstrate smaller deviations across the scanners. It may be that the bigger differences (< 1%) rather reflect registration errors and issues with partial volumes than scanner effects.

Moreover, when evaluating intra- and inter-scanner effects jointly in the two-way ANOVA test, a similar pattern was identified. The results in Figure 7 for FA, as well as those for the additional diffusion metric maps (MD, AD, RD, MK, AK, and RK), demonstrated statistically significant inter-scanner effects for most of the evaluated WM regions, while the intra-scanner effects are significant for less WM regions. When looking at the regions that demonstrated significant intra-scanner effects, there is a consistent identification of ALIC and the PLIC WM regions in all diffusion metric maps and the identification of PCR, ACR, TP, SLF, SRC, and SFOF WM regions for most of the diffusion metric maps. The identification of these specific WM regions for intra-scanner differences might be related to the size and location of those brain tracts, compared to the field homogeneity for the scanners, making the inter- and intra-scanner effects more apparent in such regions.

The scan-rescan variability observed in our study, both within and across scanners, compares well with observations previously reported for diffusion metrics in healthy volunteers (Acheson et al., 2017; Grech-Sollars et al., 2015; Kamagata et al., 2015; Prohl et al., 2019; Shahim et al., 2017; Zhou et al., 2018). Literature on harmonization multi-site dMRI datasets has demonstrated a non-uniform variability across the structure of the brain white matter, with changes up to 5% in diffusion metric maps of major pathways in the brain (Nencka et al., 2018; Pinto et al., 2020; Vollmar et al., 2010). Moreover, multi-scanner multi-shell diffusion data has been investigated in studies using traveling subjects (Kurokawa et al., 2021). In those studies, it was identified that inter-scanner systematic differences represent a risk of bias in directly comparing datasets obtained from different scanners. When evaluating the coefficient of variation between subjects, an inter-scanner variation of 7.7% was obtained in the WM, which is similar to the results presented on Figure 6B, in which significant inter-scanner variation of < 5% were identified. Such inter-scanner variabilities in diffusion metrics maps are in the same order as biological changes due to pathologies.

In previous studies, intra-scanner variations have been demonstrated to exhibit a degree of variability comparable to the differences observed between patients with mild and moderate traumatic brain injuries, specifically in the diffusion metric maps of the corpus callosum (Kumar et al., 2009). This suggests that the inherent fluctuations within a single scanner can mirror the degree of variability typically seen when comparing clinical populations with differing levels of brain injury severity. Such similarities highlight the potential challenges in distinguishing between physiological or pathological changes and technical artifacts in neuroimaging studies. Consequently, it is critical to carefully consider intra-scanner variations when interpreting diffusion metrics, particularly in clinical populations where subtle differences in brain structure may be present. Moreover, for dementia patients compared to controls, there has been evidence that longitudinal disease-related changes are similar to site variabilities (Mahoney et al., 2015). Based on consistent findings, inter-scanner diffusion MRI measurement presents variability challenges in large, multi-center studies (Tax et al., 2019).

The intra- and inter-scanner variations in the non-harmonized diffusion metric maps that were found in the current study align with these findings and demonstrate the need for data harmonization to remove the “TestRetest” and “Scanner” effects, so as to improve data comparability and foster the detection of true biological effects. The evaluated diffusion metrics (FA, MD, AD, RD, MK, AK, and RK) demonstrated a reduction of scanner effect after harmonization. This agrees with results of previous studies applying NeuroCombat to a cross-sectional dataset (Fortin et al., 2017) and LongCombat to both cross-sectional and longitudinal diffusion data (Beer et al., 2020; Richter et al., 2022). We extended the evaluations from previous studies by implementing NeuroCombat and LongCombat harmonization methods ROI-based and voxel-wise in the test-retest cross-scanner B-Q MINDED dataset.

When evaluating the intra-scanner effects after harmonization (Figures 9, 10), it is noticeable that the ROI- and voxel-wise implementations of NeuroCombat were effective in removing the TestRetest significant factor on the diffusion metric maps, while NeuroCombatScanner and LongCombat implementations failed to remove the significance in the intra-scanner differences for all WM regions. NeuroCombatScanner and LongCombat offer different strategies for harmonizing diffusion MRI data across scanners and sessions, but their handling of intrascanner variation, particularly in test-retest scenarios, can lead to unfulfilling intra-scanner harmonizing outcomes. NeuroCombatScanner combines test and retest data from the same scanner while including subject ID as a biological covariate. However, if the model treats test and retest scans from the same individual as independent—essentially as different subjects—then it may fail to account for within-subject variation, potentially misattributing intrascanner effects to inter-subject differences. As a result, true test-retest variability that arises from non-biological factors (e.g., subject motion, fatigue, scanner drift) might not be effectively removed. In contrast, LongCombat is explicitly designed to model repeated measurements on the same subject across time points. It assumes that intra-scanner differences reflect biological changes occurring between sessions rather than technical noise or behavioral variability. Consequently, LongCombat does not correct for such intra-subject differences, under the assumption that they are biologically meaningful—an assumption that may not always hold in short-term or test-retest studies where behavioral or scanner-related factors may dominate.

Moreover, the results of our evaluation of the inter-scanner effects after the implementation of the harmonization algorithms (Figures 11, 12), demonstrate that the ROI- and voxel-wise implementation of LongCombat were optimal in the removal of the significant scanner effects. In comparison, the NeuroCombat and NeuroCombatScanner approaches removed the scanner factor for some WM regions, but not all, demonstrating to be sub-optimal in comparison to LongCombat for the diffusion dataset under study. Both NeuroCombat methods yielded almost identical results on all diffusion metric maps.

The evaluation of within-subject variability using CVws (Figure 13) provided insight into how harmonization impacts measurement consistency in diffusion MRI. Our results demonstrated that both NeuroCombat and NeuroCombatScanner generally reduced or maintained CVws in intra-scanner comparisons, particularly when applied ROI-wise. This suggests that these harmonization approaches are effective at preserving within-subject consistency across repeated measures—a critical requirement for robust diffusion studies (Fortin et al., 2017; Pinto et al., 2020). In contrast, LongCombat appeared to increase variability, particularly at the voxel level - a somewhat counterintuitive and concerning outcome for a harmonization method. This effect may stem from LongCombat’s more aggressive modeling of longitudinal variance, which, while designed to account for within-subject changes over time, could inadvertently amplify noise when applied to datasets where minimal biological change is expected between sessions. These findings underscore the importance of aligning harmonization strategies with the underlying characteristics of the data, especially in studies where stability across timepoints is anticipated. Moreover, these findings are consistent with prior studies noting that overly aggressive harmonization can inadvertently amplify local noise or suppress meaningful signal variation (Beer et al., 2020). Notably, the fornix (FX) emerged as a region with high sensitivity to harmonization effects, likely reflecting its small size and higher susceptibility to registration and partial volume errors.

Maintaining between-subject biological variability is crucial when applying harmonization to datasets used in group-level comparisons or machine learning applications. Our CVbs analysis (Figure 14) showed that all harmonization methods preserved between-subject variance across scanners, with only minor deviations from the original data, especially in the voxel-wise results. This outcome supports the intended goal of methods like ComBat—to remove unwanted scanner-related variance while preserving biologically meaningful differences (Fortin et al., 2018). The subtle changes in regions like the fornix (FX), left tapetum (TP-L), and left cingulum hippocampus (CH-L) suggest some regional susceptibility to harmonization-induced distortions, potentially due to anatomical variability or low SNR in these tracts. However, the minimal impact across most regions supports the use of ComBat-based harmonization for studies requiring reliable inter-individual comparisons in healthy populations with limited biological variance.

The intraclass correlation coefficient (ICC) provides a composite measure of both within- and between-subject variability, and is widely used to assess test-retest reliability in neuroimaging (Luque Laguna et al., 2020). In our study (Figure 15), ICC values remained stable or slightly decreased following harmonization with NeuroCombat and NeuroCombatScanner, suggesting that these methods effectively retained the reliability of individual differences across sessions and scanners. However, LongCombat consistently reduced ICCs, particularly in inter-scanner comparisons, which may reflect its stronger assumptions about biological change over time—assumptions less applicable in short-interval test-retest designs. The pronounced drop in ICC within the fornix (FX) under LongCombat further highlights this region’s vulnerability and aligns with prior reports that some deep white matter tracts are more prone to measurement instability (Bastiani et al., 2019). Overall, these results suggest that while ComBat-based harmonization improves data comparability, careful consideration of region-specific effects and harmonization model assumptions is essential, especially in multi-site and longitudinal designs.

The six evaluated Combat-based harmonization algorithms (NeuroCombat, NeuroCombatScanner, and LongCombat, ROI-based and voxel-wise) demonstrated comparable performance across different research contexts, with their effectiveness largely dependent on the specific research question at hand. From a practical standpoint, their implementation can be considered straightforward, particularly from a workflow perspective, as these algorithms are associated with a relatively low risk of technical errors. Based on our comprehensive evaluation, we recommend the use of NeuroCombat for cross-sectional studies that involve multi-site and/or multi-scanner data, as well as for intra-scanner test-retest evaluations, where harmonization of scanner variability is crucial. In contrast, LongCombat is more suited for longitudinal studies involving data collected across different sites and/or scanners, where temporal consistency and the alignment of longitudinal data are key priorities.

The strengths of our study include evaluating the same subjects for intra- and inter-scanner effects, allowing for a direct comparison of harmonization impacts. The reference point is the data collected from each subject imaged twice on the same scanner, while the comparison involves the harmonized data from the same subject scanned on two different machines. Additionally, in our study, we systematically assessed performance across a range of diffusion metrics, using both voxel- and ROI-level harmonization techniques to provide a comprehensive evaluation. Our findings indicate that the outcomes of ROI-based and voxel-wise harmonization approaches were strikingly similar, suggesting that either method can be employed effectively depending on the specific objectives of the research. However, there are key considerations that differentiate the two approaches. The voxel-wise approach, while capable of harmonizing at a finer level of detail, carries a higher risk of registration errors, particularly when applying the harmonization directly to the diffusion map. This can introduce variability that may affect the accuracy of the results. On the other hand, the ROI-based approach is generally easier to implement, as a single value can represent an entire region, which simplifies the process. However, this method does not harmonize the diffusion map at a voxel level, limiting the ability to make comparisons across different white matter (WM) regions. Given these trade-offs, we recommend that researchers choose the harmonization approach that aligns best with the specific requirements of their study. Factors such as the desired spatial resolution, the particular regions of interest, and the sensitivity to inter-subject variability should all be carefully considered when making this decision. Researchers should also weigh the potential for registration errors in the voxel-wise approach vs. the simplified implementation but reduced comparison capabilities of the ROI-based method.

Furthermore, since repeated scans of the same subject were conducted on the same day, it can be reasonably assumed that no significant biological changes occurred between the scans. As a result, any observed intra-subject variability can be attributed solely to inter-scanner effects, minimizing the potential for confounding factors related to biological fluctuations. In addition, the application of two complementary analytical methods to the diffusion data yielded consistent findings, further strengthening the reliability and validity of our conclusions. This convergence of results across different methodological approaches underscores the robustness of our analysis and supports the interpretation of the observed effects as reflective of true scanner-related variability rather than underlying biological changes. Moreover, subject-related factors can significantly influence the dMRI measurements, potentially confounding results, especially in multi-scanner studies. One key factor is subject motion, which can distort diffusion metrics by introducing artifacts that obscure the true underlying signal. First-time participants may experience heightened anxiety or nervousness during their initial scan, leading to increased motion or physiological changes that affect image quality. In contrast, during a second scan—whether on the same day or at a different time—subjects may feel more comfortable and relaxed, resulting in improved compliance and reduced motion. Conversely, the timing and duration of scanning sessions can also lead to fatigue, restlessness, or impatience, particularly if the second scan occurs later in the session or in a different scanner, potentially increasing motion or decreasing attention. These behavioral and psychological factors underscore the importance of considering subject state and scan order when designing studies and interpreting dMRI extracted data.

Our analysis has two key limitations. First, the relatively small sample size may reduce statistical power, limiting our ability to detect subtle effects of harmonization. Second, the evaluation was restricted to two MRI scanners from the same manufacturer (Siemens Healthineers), which may have constrained the range of scanner-related variability and limited the generalizability of our findings to more heterogeneous multi-site datasets. While harmonization methods aim to correct scanner-related differences, the magnitude and nature of these differences can vary substantially depending on the source. In particular, inter-scanner variability is often more pronounced when comparing scanners from different manufacturers, due to underlying differences in hardware components, such as gradient systems and RF coils, as well as proprietary image reconstruction pipelines (Lee et al., 2019). As our study did not include scanners from other vendors, the generalizability of our findings to more heterogeneous, multi-manufacturer datasets remain uncertain, and future work should explore the performance of harmonization approaches under these more challenging conditions.

The limited sample size may also have reduced the statistical power needed to identify small but meaningful effects, while the inter-scanner variability (despite using scanners of the same manufacturer) could have introduced additional differences in field homogeneity, complicating the detection of finer improvements in harmonization. These constraints underscore the need for future studies to incorporate larger, more diverse datasets and ensure greater standardization in scanner performance. Such efforts would help enhance the sensitivity and precision of results in structural data harmonization, particularly when dealing with subtle or complex neuroimaging features.

Furthermore, potential technical confounders may have influenced the measurement of diffusion metrics. One such confounder is the possibility of a mismatch between the diffusion images and the co-registered structural template, which could introduce spatial inaccuracies, thereby affecting the reliability of the diffusion measurements. Additionally, scanner drift, referring to gradual changes in scanner performance over time, was not accounted for in the data analysis across subjects. This omission may have influenced both within-scanner and across-scanner follow-up measurements in various ways, potentially leading to inconsistencies or distortions in the data. However, it is important to note that no significant scanner updates occurred during the study period, and previous research by Vos et al. (2017) and Jalnefjord et al. (2024) have suggested that the impact of scanner drift is generally minimal (2–5%) but comparable to the intra- and inter-scanner variations observed in this study. Nevertheless, future analyses would benefit from incorporating strategies to correct for scanner drift and ensure more precise alignment between diffusion images and structural templates. By addressing these factors, potential sources of error could be minimized, thereby enhancing the accuracy and robustness of the findings.

Additionally, it is essential to interpret the performance of LongCombat within the appropriate context. Most cross-sectional studies involve only a single scan per subject, making the application of LongCombat unfeasible in such cases. While many longitudinal studies provide either across-scanner or within-scanner data for each participant, it is relatively rare to find datasets that include both types of data, which could potentially limit the effectiveness of LongCombat in those contexts. In contrast, the other ComBat-based algorithms tested do not rely on repeated measures per subject, suggesting that their performance estimates may be more broadly applicable to a wider range of study designs.

It is important to note that the B-Q MINDED dataset comprises healthy adult participants with a narrow age range and balanced sex distribution. While this controlled design is well-suited for assessing scanner-related variability, it inherently limits natural biological variation. Consequently, the biological covariate preservation analysis revealed mostly non-significant effects, with only one age-by-sex interaction persisting after harmonization. This result is expected given the cohort’s demographic homogeneity and underscores a key limitation: the dataset is not optimal for evaluating the preservation of complex biological signals, such as disease effects, in harmonization workflows.

## 5 Conclusion

In conclusion, the structural integrity of brain white matter can be effectively assessed using quantitative diffusion metric maps derived from both intra- and inter-scanner datasets. Given the inherent variability across scanners and imaging protocols, it is critical to explore whether harmonization of diffusion metrics can improve the accuracy and consistency of evaluations across different scanners. This study provides a comprehensive assessment of ComBat-based harmonization algorithms designed to minimize intra- and inter-scanner variability in diffusion metric maps (including FA, MD, AD, RD, MK, AK, and RK). To this end, we evaluated the following harmonization strategies: (1) NeuroCombat voxel, (2) NeuroCombat ROI, (3) NeuroCombatScanner voxel, (4) NeuroCombatScanner ROI, (5) LongCombat voxel, and (6) LongCombat ROI. These methods were assessed using the B-Q MINDED dataset, a benchmark MRI resource comprising diffusion data from 38 age- and sex-matched healthy participants. However, given the complexity of scanner-related variability and the diversity of harmonization approaches, we refrain from drawing a definitive conclusion regarding the superiority of any single method. This is primarily due to the absence of a ground truth and the sensitivity of performance metrics to specific preprocessing choices and evaluation frameworks. Instead, we emphasize the value of this dataset in facilitating further research and benchmarking, acknowledging that harmonization performance may depend on specific use cases and analytical goals. Our assessment utilized the B-Q MINDED dataset, a benchmark MRI database comprising diffusion data from 38 healthy subjects, matched by age and sex, providing a robust foundation for examining harmonization methods.

The results of this study demonstrated that both NeuroCombat and LongCombat algorithms, when applied in either ROI-based or voxel-wise configurations, significantly reduce variability in the diffusion metrics, bringing them to levels comparable to the inter-subject scan-rescan variability observed in repeated scans using the same scanner and protocol. Furthermore, our analysis highlighted the considerable impact of inter-subject variability as a major contributor to overall variability in the diffusion maps, underscoring the need for harmonization techniques that account for both technical and biological variability.

The broad evaluation of these harmonization approaches provides valuable insights for researchers seeking to harmonize diffusion data across multiple sites and scanners. In particular, the findings will aid in selecting the most appropriate harmonization methods for future multi-site studies, including those involving traveling subjects. This is of particular importance for clinical research, where the application of these algorithms can play a critical role in assessing brain structural changes associated with various neurological pathologies, such as traumatic brain injury, dementia, and neurodegenerative disorders. By minimizing the confounding effects of scanner-specific biases, harmonization techniques like those evaluated in this study promise to enhance the reliability and reproducibility of diffusion-based biomarkers.

Ultimately, the implementation of these harmonization algorithms will not only improve the accuracy of cross-site comparisons but also facilitate the pooling of data from multiple sites, enabling large-scale studies that could significantly advance our understanding of brain health and disease. Moreover, these improvements in data consistency are expected to contribute to more robust clinical decision-making and better patient outcomes in the context of brain-related disorders.

## Data Availability

The datasets presented in this study can be found in an online repository. The name of the repository and access can be found below: Zenodo: https://zenodo.org/records/6473268.

## Footnotes

1
https://zenodo.org/records/6473268

2
https://github.com/Jfortin1/neuroCombat_Rpackage

3
https://github.com/jcbeer/longCombat
